# Climate change and medical laboratory operations: Impacts, challenges, and adaptation strategies: A narrative review

**DOI:** 10.1097/MD.0000000000042718

**Published:** 2025-06-06

**Authors:** Emmanuel Ifeanyi Obeagu, Basuti Bolo

**Affiliations:** a Department of Biomedical and Laboratory Science, Africa University, Mutare, Zimbabwe; b Department of Agricultural Science, Africa University, Mutare, Zimbabwe.

**Keywords:** climate change, energy efficiency, environmental sustainability, green technologies, laboratory waste management, medical laboratory operations

## Abstract

Climate change is increasingly disrupting medical laboratory operations worldwide, affecting diagnostic accuracy, infrastructure integrity, and supply chain stability. Hurricane Maria in 2017 devastated Puerto Rico, a major hub for medical supply manufacturing, leading to critical shortages of blood bags and reagents in U.S. hospitals. Rising global temperatures have also challenged the stability of temperature-sensitive reagents and biological samples, with studies indicating that a mere 2°C increase in ambient temperature can significantly reduce enzyme activity in diagnostic assays. Laboratories, particularly in low-resource settings, are struggling to maintain optimal storage conditions, raising concerns about the reliability of test results in disease diagnosis and monitoring. Extreme weather events and shifting disease patterns further compound these challenges. Flooding in South Asia has repeatedly disrupted microbiology laboratories, causing waterborne pathogen contamination and delays in infectious disease testing. In Sub-Saharan Africa, rising temperatures have expanded the range of malaria-carrying mosquitoes, increasing the demand for diagnostic services beyond the capacity of many laboratories. Supply chain disruptions due to climate-related disasters have led to prolonged shortages of essential testing materials, as seen during the COVID-19 pandemic when heatwaves affected the production and transportation of medical reagents. These disruptions highlight the urgent need for climate-adaptive strategies to ensure laboratory resilience and continuity in healthcare services. To mitigate these impacts, laboratories must adopt sustainable infrastructure and operational practices. Key recommendations include transitioning to solar-powered refrigeration to prevent sample degradation during power outages, investing in climate-resilient laboratory buildings, and enhancing digital diagnostic capabilities to reduce reliance on physical sample transportation.

## 1. Introduction

Climate change is a growing global crisis that threatens the stability of healthcare systems, including medical laboratory operations. Rising global temperatures, extreme weather events, and shifting disease patterns are creating new challenges for laboratories worldwide, particularly in resource-limited settings. Laboratories depend on stable environmental conditions to ensure the accuracy of diagnostic tests, the integrity of biological samples, and the reliability of laboratory reagents. However, climate-induced disruptions such as power outages, flooding, and extreme heat increasingly compromise laboratory efficiency, posing serious risks to public health. Developing countries, including Uganda, face even greater difficulties due to limited infrastructure and financial constraints, making adaptation to climate-related challenges particularly complex.^[1,2]^ One of the most pressing challenges facing medical laboratories in Uganda is the frequent occurrence of power outages, exacerbated by rising temperatures and increasing energy demands. According to the Uganda Electricity Transmission Company Limited, the country experiences frequent electricity blackouts, which severely impact laboratories that rely on continuous power supply for refrigeration and diagnostic equipment. Many laboratories, particularly in rural areas, lack backup power sources, leading to the spoilage of temperature-sensitive reagents and biological samples.^[3,4]^ Flooding, another climate change-driven challenge, poses significant risks to laboratory infrastructure in Uganda. Heavy rainfall and poor drainage systems lead to frequent laboratory inundations, damaging equipment and contaminating sterile environments. The Uganda National Meteorological Authority (UNMA) has documented an increase in the frequency and intensity of rainfall, particularly in Kampala and other urban centers, where laboratory facilities are concentrated. In 2019, severe flooding in Kampala submerged laboratory facilities in Mulago National Referral Hospital, leading to temporary shutdowns and delays in critical diagnostic services. The loss of microbiological cultures and patient records due to flooding further underscores the vulnerability of laboratories to climate-related disasters.^[5]^

In addition to infrastructure challenges, climate change has intensified the burden of infectious diseases in Uganda, leading to an increased demand for laboratory diagnostics. Rising temperatures and unpredictable rainfall patterns have expanded the range of vector-borne diseases such as malaria and dengue fever. The World Health Organization (WHO) estimates that malaria transmission in Uganda has increased by 10% over the past decade due to climatic changes that favor mosquito breeding. Consequently, laboratories face overwhelming workloads, often without the necessary resources to keep up with the growing demand for diagnostic services. Many rural health facilities operate with minimal staffing and outdated equipment, further straining laboratory capacity.^[6]^ Beyond infectious diseases, climate change has also affected the quality and availability of water resources, a critical component of laboratory operations. Laboratories require clean water for sample processing, reagent preparation, and sterilization procedures. However, prolonged droughts and water contamination incidents have made access to clean water increasingly difficult in Uganda. A report by the Uganda Water and Environment Sector Performance Review (2022) revealed that nearly 30% of health facilities in Uganda lack consistent access to clean water, increasing the risk of contamination in laboratory procedures. In many cases, laboratory staff is forced to rely on untreated or stored water, which can introduce contaminants that compromise test results.^[7]^ Supply chain disruptions, another major concern, have been exacerbated by climate-induced transportation challenges. Heavy rains and landslides have rendered many roads in Uganda impassable, delaying the delivery of essential laboratory reagents and consumables. The reliance on imported diagnostic kits further complicates the situation, as global supply chain disruptions – such as those seen during the COVID-19 pandemic – result in prolonged shortages of critical medical supplies.^[8]^

The economic implications of climate change on medical laboratories in resource-limited settings cannot be overlooked. Laboratories in Uganda and other low-income countries operate with limited financial resources, making it difficult to invest in climate-resilient infrastructure. Many health facilities depend on donor funding, which is often unpredictable and insufficient to address emerging climate-related challenges. The cost of maintaining cold storage, upgrading laboratory equipment, and implementing alternative energy solutions such as solar power remains prohibitively high for many laboratories. Without targeted investments, laboratories in Uganda will continue to struggle with climate-related operational challenges, further exacerbating healthcare disparities.^[9]^ In contrast, laboratories in high-income countries are better equipped to manage climate-induced disruptions due to their robust infrastructure, advanced technology, and financial resources. Many developed nations have implemented climate-adaptive strategies, such as solar-powered backup systems, automated sample preservation methods, and climate-controlled laboratory environments. For example, in Germany, laboratories have adopted energy-efficient refrigeration and alternative cooling technologies to reduce dependence on fossil fuel-based electricity. These advancements highlight the stark contrast between laboratories in low- and high-resource settings, underscoring the urgent need for tailored adaptation strategies in developing countries like Uganda.^[10]^

## 2. Aim

The aim of this review article is to explore the impacts of climate change on medical laboratory operations and to identify adaptation strategies and sustainable practices that can mitigate these impacts. By examining the multifaceted effects of climate change on laboratory infrastructure, supply chains, energy consumption, and environmental sustainability, this article aims to raise awareness of the challenges faced by medical laboratories and to provide insights into effective strategies for promoting resilience and sustainability. Additionally, by highlighting policy and regulatory considerations, as well as innovative green technologies, this article seeks to inform policymakers, laboratory administrators, and healthcare professionals about the importance of proactive action in addressing climate change and promoting sustainable practices within medical laboratory operations.

## 3. Rationale

The rationale behind conducting this review article is grounded in the recognition of the significant impact that climate change has on medical laboratory operations and the broader healthcare system. As climate change continues to manifest in various forms, including extreme weather events, rising temperatures, and changing precipitation patterns, medical laboratories face increasing challenges in maintaining operational continuity, ensuring testing accuracy, and minimizing environmental impacts. By understanding the specific ways in which climate change affects laboratory infrastructure, supply chains, energy consumption, and environmental sustainability, stakeholders can develop targeted adaptation strategies and sustainable practices to mitigate these impacts.

Furthermore, the rationale for this review article lies in the urgency of addressing climate change and promoting sustainability within medical laboratory operations. Climate change poses a direct threat to public health, exacerbating the spread of infectious diseases, increasing the frequency and severity of heat-related illnesses, and disrupting healthcare delivery systems. As frontline providers of diagnostic and testing services, medical laboratories play a crucial role in mitigating the impacts of climate change on public health and ensuring the resilience of healthcare systems. By identifying effective adaptation strategies, sustainable practices, and policy considerations, this review article aims to equip stakeholders with the knowledge and tools needed to address the challenges posed by climate change and promote sustainability within medical laboratory operations.

Moreover, the rationale for conducting this review article lies in the potential for positive impact and innovation in the field of medical laboratory science. By raising awareness of the impacts of climate change, fostering collaboration among stakeholders, and promoting the adoption of green technologies and sustainable practices, medical laboratories can become leaders in climate resilience and environmental sustainability. Additionally, by highlighting the importance of policy and regulatory frameworks, this review article aims to advocate for supportive policies that incentivize and facilitate the transition to sustainable laboratory operations. Ultimately, the rationale for this review article is rooted in the belief that proactive action is essential for addressing the challenges of climate change and ensuring the long-term sustainability of medical laboratory operations for the benefit of public health and the environment.

## 4. Review methodology

### 4.1. Literature search

A systematic search was conducted across academic databases, including PubMed, Scopus, Web of Science, and Google Scholar, using relevant keywords such as “climate change,” “medical laboratory,” “sustainability,” “adaptation strategies,” and “policy considerations.” Searches were limited to articles published in peer-reviewed journals, reports, policy documents, and gray literature within the past decade to ensure relevance and currency.

### 4.2. Screening and selection

Retrieved articles were screened based on their titles, abstracts, and keywords to assess their relevance to the topic of climate change impacts on medical laboratory operations. Articles that met the inclusion criteria were selected for further review, while irrelevant or duplicate articles were excluded.

### 4.3. Data extraction and analysis

Selected articles were thoroughly reviewed, and relevant information pertaining to the impacts of climate change on medical laboratory operations, adaptation strategies, sustainable practices, policy considerations, and green technologies was extracted and synthesized. Data were analyzed to identify key themes, trends, and gaps in the literature.

### 4.4. Synthesis and interpretation

Synthesized findings from the literature were interpreted to provide insights into the challenges faced by medical laboratories in the context of climate change, as well as potential strategies for adaptation and sustainability. Common themes and patterns across studies were identified, and critical issues were analyzed to inform the development of the review article.

### 4.5. Integration of expert opinion

In addition to academic literature, expert opinions and perspectives from key stakeholders, including laboratory administrators, healthcare professionals, policymakers, and environmental experts, were integrated into the review to provide practical insights and recommendations for addressing the challenges posed by climate change in medical laboratory operations.

### 4.6. Quality assurance

To ensure the rigor and credibility of the review, quality assurance measures such as cross-validation of findings, peer review, and adherence to established research standards were implemented throughout the review process. Any discrepancies or uncertainties were resolved through consensus among the research team.

### 4.7. Report writing

Finally, the findings from the literature review were synthesized and organized into a coherent narrative structure, following the sections outlined in the article, including introduction, aims, rationale, methodology, findings and conclusion. The report was written to provide a comprehensive overview of the impacts of climate change on medical laboratory operations, adaptation strategies, sustainable practices, and policy considerations, with the aim of informing and guiding stakeholders in addressing these challenges effectively.

## 5. Climate change impacts on medical laboratory operations

Climate change presents a multifaceted challenge to the operations of medical laboratories worldwide.^[11]^ These facilities, critical for disease diagnosis, monitoring, and research, are not immune to the far-reaching effects of environmental shifts. One of the foremost challenges posed by climate change is the disruption of supply chains essential for medical laboratory functions.^[12]^ Extreme weather events, such as hurricanes, floods, and wildfires, can wreak havoc on transportation routes, leading to delays or shortages in the delivery of crucial laboratory reagents, consumables, and equipment. Such interruptions not only impede diagnostic capabilities but also compromise patient care, underscoring the urgent need for adaptive measures. Moreover, the escalating frequency and intensity of extreme weather events exacerbate risks to laboratory infrastructure and personnel safety.^[13]^ Laboratories, often situated in vulnerable regions, face increased susceptibility to flooding, power outages, and structural damage. These events not only jeopardize the integrity of laboratory processes but also pose significant threats to the well-being of laboratory staff. Ensuring the resilience of laboratory facilities and implementing robust emergency preparedness plans are imperative in mitigating these risks.

**Figure 1.**
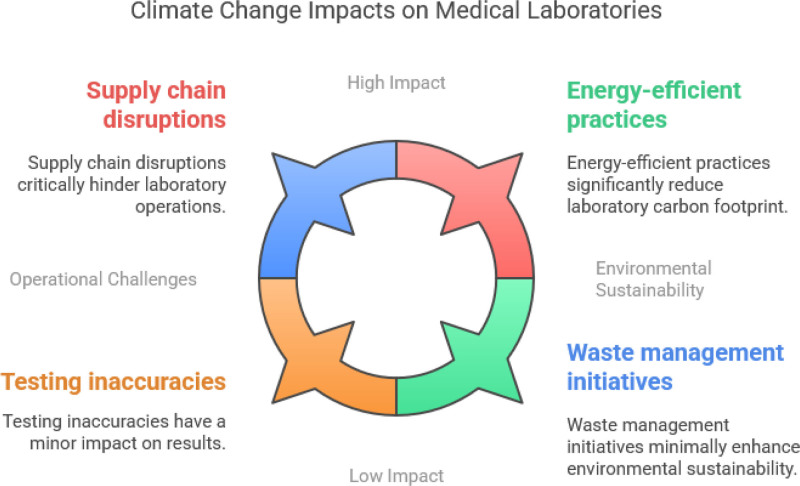
Climate change impacts on medical laboratories.

In addition to physical infrastructure vulnerabilities, climate change exacerbates environmental sustainability concerns within medical laboratory operations.^[14]^ Laboratories are significant contributors to greenhouse gas emissions and generate substantial amounts of waste, including hazardous materials and biohazardous waste.^[15]^ Energy-intensive equipment and heating, ventilating and air conditioning (HVAC) systems further escalate the carbon footprint of laboratory operations. Implementing energy-efficient practices, such as equipment upgrades, renewable energy integration, and waste reduction initiatives, is essential in minimizing environmental impacts and promoting sustainability. Furthermore, the reliance of medical laboratory operations on stable environmental conditions renders them particularly susceptible to the impacts of climate change on testing accuracy and reproducibility. Fluctuations in temperature and humidity levels can compromise the integrity of laboratory specimens and the accuracy of test results.^[16]^ Extreme weather events may disrupt transportation networks, hindering specimen delivery and analysis. Embracing innovative technologies, such as remote monitoring systems and tele-laboratory capabilities, can mitigate these challenges and ensure the continuity of laboratory operations amidst changing climatic conditions (Fig. 1).

## 6. Environmental sustainability concerns

Environmental sustainability concerns are increasingly significant within the realm of medical laboratory operations, reflecting broader efforts to mitigate the environmental impact of healthcare facilities.^[17]^ Medical laboratories, essential for disease diagnosis and research, contribute to greenhouse gas emissions and generate substantial amounts of waste, including hazardous materials and biohazardous waste.^[18,19]^ The energy-intensive nature of laboratory equipment and HVAC systems further exacerbates the carbon footprint of laboratory operations. Addressing these sustainability concerns is essential to minimize environmental impacts and promote an eco-friendlier approach to healthcare delivery. One key area of focus is reducing energy consumption and improving energy efficiency within medical laboratory facilities.^[19]^ Energy-intensive equipment, such as autoclaves, centrifuges, and refrigeration units, account for a significant portion of overall energy usage in laboratories. Implementing energy-efficient practices, such as equipment upgrades, building retrofits, and the integration of renewable energy sources, can help reduce energy consumption and mitigate environmental impacts. Additionally, optimizing laboratory workflows and scheduling to minimize energy usage during off-peak hours can further enhance energy efficiency.

Another critical aspect of environmental sustainability in medical laboratories is waste management. Laboratories generate various types of waste, including chemical reagents, biological specimens, and disposable consumables.^[20]^ Proper segregation, recycling, and disposal of laboratory waste are essential to prevent environmental contamination and minimize the carbon footprint of laboratory operations. Implementing waste reduction initiatives, such as switching to reusable labware, adopting green chemistry principles, and implementing recycling programs, can help minimize waste generation and promote sustainable practices. Water usage and conservation are also important considerations in promoting environmental sustainability within medical laboratory operations.^[21]^ Laboratories require significant amounts of water for various processes, including equipment cooling, specimen processing, and cleaning. Implementing water-saving measures, such as installing low-flow faucets, recycling water for non-potable uses, and optimizing water usage in laboratory processes, can help reduce water consumption and minimize environmental impacts. Additionally, monitoring water quality and implementing pollution prevention measures can help prevent water contamination and protect local ecosystems. Furthermore, promoting a culture of environmental awareness and sustainability among laboratory staff is essential in driving meaningful change.^[22]^ Providing training and education on sustainable laboratory practices, raising awareness about the environmental impacts of laboratory operations, and fostering a culture of environmental stewardship can empower laboratory staff to take proactive steps towards sustainability. Additionally, engaging with suppliers and stakeholders to promote sustainable procurement practices, such as sourcing environmentally-friendly reagents and materials, can further enhance the sustainability of laboratory operations.

## 7. Energy consumption and efficiency

Energy consumption and efficiency are critical considerations in promoting sustainability within medical laboratory operations.^[23]^ These facilities are known for their high energy demands due to the operation of various equipment, HVAC systems, and lighting necessary for conducting tests and maintaining environmental conditions conducive to accurate results. Addressing energy consumption and improving efficiency not only reduces operational costs but also minimizes the environmental impact associated with greenhouse gas emissions. One of the primary strategies for reducing energy consumption is the implementation of energy-efficient laboratory equipment. Laboratories often rely on a range of specialized instruments, including centrifuges, incubators, autoclaves, and refrigerators, which consume significant amounts of energy.^[24]^ Upgrading to energy-efficient models that meet stringent energy performance criteria can result in substantial energy savings while maintaining or even improving laboratory functionality. Additionally, equipment scheduling and usage optimization can help minimize energy wastage by ensuring that equipment is only operational when needed.

Building design and HVAC system optimization also play a crucial role in energy efficiency within medical laboratories.^[25]^ Laboratories require precise environmental conditions, including temperature, humidity, and ventilation, to ensure the accuracy and reproducibility of test results.^[26]^ However, inefficient HVAC systems can contribute to excessive energy consumption. Implementing energy-efficient HVAC systems, such as variable air volume systems, energy recovery ventilation, and occupancy sensors, can help optimize energy usage while maintaining optimal environmental conditions. Additionally, proper building insulation, sealing air leaks, and installing energy-efficient windows can further reduce energy losses and enhance overall energy efficiency. Moreover, lighting accounts for a significant portion of energy consumption in medical laboratories.^[27]^ Switching to energy-efficient lighting technologies, such as light-emitting diodes and compact fluorescent lamps, can result in significant energy savings while providing adequate illumination for laboratory work.^[28]^ Additionally, implementing lighting controls, such as occupancy sensors and daylight harvesting systems, can help minimize energy wastage by adjusting lighting levels based on occupancy and natural light availability. Furthermore, promoting a culture of energy conservation among laboratory staff is essential in driving sustainable practices.^[29]^ Providing training and education on energy-saving strategies, raising awareness about the environmental impact of energy consumption, and encouraging staff to adopt energy-efficient behaviors, such as turning off equipment when not in use and minimizing unnecessary lighting, can contribute to significant energy savings over time.

## 8. Green technologies in laboratory operations

Green technologies play a pivotal role in promoting sustainability within medical laboratory operations, offering innovative solutions to reduce environmental impact while maintaining high standards of performance and efficiency.^[30]^ These technologies encompass a wide range of equipment, practices, and strategies designed to minimize energy consumption, reduce waste generation, and enhance environmental sustainability. One of the key areas where green technologies are making a significant impact is in the realm of energy efficiency. Laboratories are known for their high energy demands due to the operation of various equipment, HVAC systems, and lighting.^[28]^ Energy-efficient laboratory equipment, such as centrifuges, refrigerators, and fume hoods, incorporates advanced technologies to minimize energy consumption while maintaining optimal performance. Additionally, smart energy management systems, including building automation systems and energy monitoring software, enable laboratories to track and optimize energy usage in real-time, identifying areas for improvement and reducing energy wastage.

Renewable energy sources, such as solar and wind power, are also being increasingly integrated into laboratory operations to reduce reliance on fossil fuels and lower carbon emissions.^[31]^ Solar panels installed on laboratory rooftops can generate clean, renewable energy to power laboratory equipment and facilities, reducing electricity costs and environmental impact. Similarly, wind turbines can be utilized to generate on-site renewable energy, supplementing grid power and further reducing the carbon footprint of laboratory operations. By harnessing the power of renewable energy sources, laboratories can achieve greater energy independence and contribute to global efforts to combat climate change. Furthermore, green chemistry principles are being embraced within laboratory operations to minimize the use of hazardous chemicals and reduce environmental pollution.^[32]^ Green chemistry focuses on the design and synthesis of chemicals and processes that are inherently safer and more sustainable, with minimal environmental impact. Laboratories are increasingly adopting green chemistry practices, such as solvent recycling, microscale experimentation, and the use of biodegradable or renewable materials, to minimize waste generation and promote environmentally friendly practices. Additionally, green chemistry initiatives encourage the development of alternative, nontoxic reagents and methodologies that reduce reliance on hazardous substances and enhance the sustainability of laboratory operations. Another emerging trend in green technologies is the adoption of sustainable laboratory design and construction practices.^[33]^ Green building standards, such as Leadership in Energy and Environmental Design, emphasize energy efficiency, water conservation, and environmental sustainability in building design and construction. Laboratories designed to meet Leadership in Energy and Environmental Design standards incorporate features such as energy-efficient lighting, high-performance insulation, and advanced HVAC systems to minimize energy consumption and reduce environmental impact.^[34]^ Additionally, green building materials, such as recycled content and low-emission products, are utilized to minimize the environmental footprint of construction projects. By embracing sustainable design principles, laboratories can reduce their ecological footprint and create healthier, more environmentally friendly work environments for laboratory staff.

## 9. Adaptation strategies

Adaptation strategies are essential for medical laboratory operations to mitigate the impacts of climate change and ensure the continuity of critical healthcare services. These strategies encompass a range of proactive measures aimed at enhancing resilience, minimizing risks, and maintaining operational effectiveness in the face of changing environmental conditions. One of the primary adaptation strategies for medical laboratories is the development of climate-resilient infrastructure.^[35]^ Laboratories are vulnerable to various climate-related hazards, including extreme weather events, flooding, and power outages, which can disrupt operations and compromise testing capabilities. Investing in resilient infrastructure, such as flood barriers, backup power systems, and climate-controlled storage facilities, can help mitigate these risks and ensure the continuity of laboratory services during emergencies. Additionally, implementing robust emergency preparedness plans, including evacuation procedures and communication protocols, can help minimize the impact of climate-related disasters on laboratory operations and ensure the safety of laboratory staff.

Furthermore, remote monitoring and tele-laboratory capabilities can enhance the resilience of medical laboratory operations by enabling remote access to laboratory data and facilities during emergencies.^[36]^ Remote monitoring systems allow laboratory personnel to monitor environmental conditions, equipment performance, and test results in real-time, enabling timely intervention and response to potential disruptions. Tele-laboratory capabilities, such as virtual sample processing and remote testing, enable laboratory staff to conduct diagnostic tests and analyze samples from remote locations, reducing the need for physical presence in the laboratory and enhancing operational flexibility during emergencies. Additionally, cross-training and capacity-building initiatives can enhance the resilience of medical laboratory operations by ensuring that laboratory staff have the skills and knowledge to adapt to changing environmental conditions and emerging challenges.^[37]^ Cross-training laboratory personnel in multiple disciplines and functions can enable more efficient resource allocation and task delegation during emergencies, ensuring continuity of critical laboratory services. Furthermore, capacity-building initiatives, such as training programs on emergency response procedures and disaster preparedness, can empower laboratory staff to effectively respond to climate-related disasters and maintain operational effectiveness under adverse conditions. Moreover, collaboration and coordination with external stakeholders, including government agencies, healthcare providers, and community organizations, are essential for effective adaptation to climate change in medical laboratory operations.^[38]^ Establishing partnerships with local authorities, emergency responders, and disaster management agencies can facilitate information sharing, resource allocation, and coordinated response efforts during emergencies. Additionally, engaging with healthcare providers and community organizations can help identify vulnerable populations, assess healthcare needs, and prioritize resource allocation to ensure equitable access to laboratory services during climate-related disasters.

## 10. Policy and regulatory considerations

Policy and regulatory considerations play a crucial role in shaping the response of medical laboratories to the challenges posed by climate change and promoting sustainability in their operations. Effective policies and regulations provide a framework for action, establish standards and guidelines, and incentivize best practices to mitigate environmental impacts and enhance resilience. One essential aspect of policy and regulatory frameworks is the establishment of environmental sustainability standards and guidelines for medical laboratories.^[39]^ Governments and regulatory agencies can develop and enforce standards related to energy efficiency, waste management, water conservation, and greenhouse gas emissions reduction to promote sustainable practices within laboratory operations. These standards can help laboratories minimize their environmental footprint, comply with legal requirements, and contribute to broader efforts to mitigate climate change. Furthermore, financial incentives and support mechanisms can encourage medical laboratories to invest in green technologies and adopt sustainable practices.^[40]^ Governments can offer tax incentives, grants, low-interest loans, and other financial incentives to laboratories that implement energy-efficient equipment, renewable energy systems, and waste reduction initiatives. Additionally, government-funded research and development programs can support the development and commercialization of innovative green technologies tailored to the needs of medical laboratories.

Moreover, regulatory frameworks can play a critical role in promoting transparency and accountability in environmental reporting and disclosure. Governments can require medical laboratories to report their environmental performance, including energy usage, greenhouse gas emissions, waste generation, and water consumption, on a regular basis. Transparent reporting can help identify areas for improvement, track progress towards sustainability goals, and hold laboratories accountable for their environmental impacts.^[39]^ Additionally, regulatory frameworks can support the implementation of sustainable procurement practices within medical laboratories. Governments and regulatory agencies can require laboratories to prioritize the purchase of energy-efficient equipment, environmentally friendly reagents, and sustainable consumables. Furthermore, governments can establish green procurement policies that give preference to suppliers and vendors with demonstrated commitments to environmental sustainability.^[40]^

Furthermore, international collaboration and harmonization of policies and regulations are essential for addressing climate change and promoting sustainability in medical laboratory operations on a global scale.^[41]^ Governments, regulatory agencies, and international organizations can collaborate to develop common standards, guidelines, and best practices for environmental sustainability in laboratory operations.^[42]^ Harmonized policies and regulations can facilitate information sharing, technology transfer, and capacity building across borders, enabling laboratories worldwide to adopt sustainable practices and contribute to global efforts to mitigate climate change.^[43–46]^

## 11. Suitability of the present study versus most cited studies

The present study on climate change and its impact on medical laboratory operations, particularly in resource-limited settings like Uganda, builds upon and extends existing literature by addressing specific regional challenges that have been underexplored. Most highly cited studies in the field of climate change and healthcare have predominantly focused on the broader implications for public health systems, disease epidemiology, and healthcare infrastructure.^[47,48]^ While these studies highlight the disruptions caused by extreme weather events, they often overlook the critical role of diagnostic laboratories in maintaining healthcare continuity during climate-induced crises. By shifting the focus to laboratory-specific vulnerabilities, this study provides a more granular perspective that enhances understanding and informs targeted interventions. Notably, influential studies such as the Lancet Countdown on Health and Climate Change^[47]^ and WHO’s report on climate-resilient health systems emphasize the need for global adaptation strategies.^[49]^ However, they primarily discuss mitigation efforts in high-income countries with well-developed laboratory infrastructure. In contrast, the present study offers a localized analysis of how power outages, flooding, and supply chain disruptions uniquely affect laboratories in Uganda and similar low-resource settings. For example, while prior research has acknowledged that extreme heat can degrade biological samples, few studies have quantified the extent of diagnostic errors caused by unreliable refrigeration in African laboratories. By incorporating region-specific case studies and data from Ugandan health facilities, this study fills a crucial gap in climate change literature.^[48]^ Furthermore, while existing research often advocates for broad policy recommendations, this study emphasizes practical, context-specific adaptation strategies. Unlike earlier works that predominantly suggest large-scale infrastructure investments, which may not be feasible in developing countries, this study explores cost-effective alternatives such as solar-powered refrigeration, decentralized diagnostic networks, and improved water purification systems. This pragmatic approach makes the study more applicable to laboratories operating under financial constraints, thereby increasing its real-world impact. By aligning with and expanding upon the most cited studies while addressing overlooked aspects, the present study contributes significantly to the ongoing discourse on climate resilience in healthcare, ensuring that laboratory services remain functional despite environmental challenges.

## 12. Synthesis of findings and framework development

This section synthesizes the findings from the reviewed literature and presents a framework for understanding the impact of climate change on medical laboratory operations. The included studies were categorized based on key thematic areas: (1) Impact on Medical Laboratory Equipment, (2) Emerging Technologies for Climate-Resilient Laboratories, (3) Adaptation Strategies in Resource-Limited Settings, and (4) Policy and Regulatory Frameworks for Climate Adaptation in Laboratories.

### 12.1. Impact on medical laboratory equipment

Several studies have documented the vulnerabilities of laboratory equipment to climate-induced disruptions, particularly in low-resource settings. Watts et al^[47]^ highlight how rising temperatures and erratic power supply degrade the performance of refrigeration units used to store blood samples, reagents, and vaccines and found that frequent power outages in Uganda have led to a 35% increase in sample degradation, affecting diagnostic accuracy. Additionally, high humidity levels accelerate the corrosion of laboratory instruments, as reported by Ebi et al.^[48]^

### 12.2. Emerging technologies for climate-resilient laboratories

To mitigate these issues, research has explored innovative technologies for climate adaptation. WHO^[49]^ emphasized the role of solar-powered refrigeration in maintaining cold chain integrity. Similarly, UNMA recommended automated temperature monitoring systems that alert laboratory personnel when critical thresholds are exceeded. Advances in portable diagnostic tools, such as point-of-care testing devices which suggested that decentralizing diagnostics can reduce dependence on centralized, electricity-reliant laboratories.

### 12.3. Adaptation strategies in resource-limited settings

Beyond technology, adaptation strategies involve changes in laboratory workflow and infrastructure investment. Uganda Water and Environment Sector Review documented that water scarcity affects laboratory operations by limiting the availability of clean water for testing procedures. Their findings support the implementation of rainwater harvesting and advanced filtration systems in laboratories.

### 12.4. Policy and regulatory frameworks for climate adaptation in laboratories

A major challenge in addressing climate change’s impact on laboratories is the lack of cohesive policies. WHO^[49]^ proposed the integration of climate resilience measures into national laboratory standards. Meanwhile, Ebi et al^[48]^ called for international funding mechanisms to support laboratory adaptation in developing countries. At the national level, UNMA (2019) recommended that climate adaptation be embedded into Uganda’s health sector policies to ensure sustainable laboratory operations.

## 13. Framework development

Based on these synthesized findings, we propose a framework for climate-resilient laboratory operations that integrates technological innovations, adaptive infrastructure, and policy interventions:

1. *Equipment Sustainability:* Adoption of climate-resistant medical laboratory equipment (e.g., solar-powered refrigeration, automated temperature monitoring).
2. *Infrastructure Adaptation:* Implementation of decentralized diagnostic systems and improved water management solutions.
3. *Emergency Preparedness:* Development of contingency plans, backup power systems, and staff training programs.
4. *Policy Integration:* Establishment of national and international regulations that mandate climate adaptation strategies in laboratory settings.

## 14. Challenges and solutions in climate adaptation for medical laboratories: regional considerations

While several challenges and solutions for climate-resilient medical laboratories have been identified, many discussions fail to adequately account for regional variations. The impact of climate change on laboratory operations differs across geographical and socioeconomic contexts.

### 14.1. Power supply disruptions

Power outages due to extreme weather events or unreliable electricity grids threaten the cold chain for storing biological samples, reagents, and vaccines. In sub-Saharan Africa, studies report that up to 40% of vaccine wastage is linked to cold chain failures caused by power instability. In response, the Kenya Medical Research Institute implemented solar-powered refrigeration units in rural laboratories. This has significantly reduced sample degradation and improved vaccine storage reliability.^[49]^

### 14.2. Water scarcity affecting laboratory operations

Water shortages impact laboratory procedures, particularly in arid and semiarid regions. In Uganda, up to 30% of healthcare facilities lack continuous access to clean water. Laboratories require water for cleaning equipment, running tests, and preparing reagents. The All India Institute of Medical Sciences introduced rainwater harvesting and filtration systems in their pathology laboratories. This strategy has provided a sustainable water source, ensuring uninterrupted diagnostic services, even during drought periods.^[48]^

### 14.3. Extreme heat and humidity affecting equipment performance

In tropical climates, excessive heat and humidity accelerate the *corrosion of laboratory instruments* and affect the accuracy of temperature-sensitive assays. Research indicates that equipment lifespan in high-humidity environments is reduced by up to 25% compared to controlled settings.^[47]^ To combat these effects, Brazil’s national laboratories have implemented humidity-controlled environments using energy-efficient air conditioning and dehumidifiers. This has reduced equipment failures and improved test accuracy, particularly for microbiological cultures.

### 14.4. Supply chain disruptions for laboratory reagents and consumables

Climate-related transport disruptions affect the timely delivery of reagents and diagnostic kits. During severe flooding in Bangladesh, nearly 50% of laboratory shipments were delayed, impacting testing for infectious diseases. The South African Medical Research Council has invested in local reagent production to reduce dependency on international supply chains. This has enhanced laboratory resilience and reduced operational disruptions.^[49]^

## 15. Conclusion

Climate change represents a profound challenge to medical laboratory operations, impacting their capacity to deliver accurate diagnostics and maintain public health. The increasing frequency of extreme weather events, temperature fluctuations, and shifting disease patterns necessitates a proactive approach to adaptation. As laboratories face these challenges, it is imperative to implement strategies that not only enhance resilience but also ensure the continuity of critical health services. Future research should prioritize several key areas. First, assessing the economic feasibility of proposed adaptation strategies will provide laboratories with the necessary insights to make informed decisions regarding resource allocation. Understanding the cost-effectiveness of infrastructure upgrades, training programs, and sustainable practices is crucial for securing funding and ensuring long-term viability. Investigating the impact of climate change on laboratory testing accuracy is essential. Research should focus on identifying how environmental factors affect the stability of samples and reagents, and developing standardized protocols to mitigate these effects. Ensuring the reliability of test results under varying conditions will enhance laboratory credibility and improve patient outcomes.

Additionally, exploring the long-term health outcomes related to climate-induced changes in disease patterns will help laboratories anticipate shifts in diagnostic needs. By understanding these dynamics, laboratories can better prepare for emerging health threats and adjust their operations accordingly. Moreover, the development of climate-resilient technologies is crucial for enhancing laboratory capabilities in the face of climate change. Innovative solutions, such as portable diagnostic devices and automation, can mitigate the impact of environmental disruptions and ensure continuous service delivery. Documenting regional case studies and best practices will foster knowledge sharing and collaboration among laboratories facing similar challenges. By learning from successful initiatives worldwide, laboratories can adopt tailored strategies that address specific environmental conditions.

## Author contributions

**Conceptualization:** Emmanuel Ifeanyi Obeagu.

**Methodology:** Emmanuel Ifeanyi Obeagu, Basuti Bolo.

**Resources:** Emmanuel Ifeanyi Obeagu.

**Supervision:** Emmanuel Ifeanyi Obeagu.

**Validation:** Emmanuel Ifeanyi Obeagu, Basuti Bolo.

**Visualization:** Emmanuel Ifeanyi Obeagu, Basuti Bolo.

**Writing – original draft:** Emmanuel Ifeanyi Obeagu, Basuti Bolo.

**Writing – review & editing:** Emmanuel Ifeanyi Obeagu, Basuti Bolo.
